# Zirconia Nanoparticles Induce HeLa Cell Death Through Mitochondrial Apoptosis and Autophagy Pathways Mediated by ROS

**DOI:** 10.3389/fchem.2021.522708

**Published:** 2021-03-16

**Authors:** Yinghui Shang, Qinghai Wang, Jian Li, Haiting Liu, Qiangqiang Zhao, Xueyuan Huang, Hang Dong, Wansong Chen, Rong Gui, Xinmin Nie

**Affiliations:** ^1^ Department of Blood Transfusion, The Third Xiangya Hospital, Central South University, Changsha, China; ^2^ Department of Cardiology, The Second Hospital of Shandong University, Jinan, China; ^3^ College of Chemistry and Chemical Engineering, Central South University, Changsha, China; ^4^ Clinical Laboratory of the Third Xiangya Hospital, Central South University, Changsha, China

**Keywords:** anticancer, reactive oxygen species, autophagy, apoptosis, zirconia

## Abstract

Zirconia nanoparticles (ZrO_2_ NPs) are commonly used in the field of biomedical materials, but their antitumor activity and mechanism is unclear. Herein, we evaluated the anti-tumor activity of ZrO_2_ NPs and explored the anti-tumor mechanism. The results of *in vitro* and *in vivo* experiments showed that the level of intracellular reactive oxygen species (ROS) in HeLa cells was elevated after ZrO_2_ NPs treatment. Transmission electron microscopy (TEM) showed that after treatment with ZrO_2_ NPs, the mitochondria of HeLa cells were swollen, accompanied with the induction of autophagic vacuoles. In addition, flow cytometry analysis showed that the apoptotic rate of HeLa cells increased significantly by Annexin staining after treatment with ZrO_2_ NPs, and the mitochondrial membrane potential (MMP) was reduced significantly. The proliferation of HeLa cells decreased as indicated by reduced Ki-67 labeling. In contrast, TUNEL-positive cells in tumor tissues increased after treatment with ZrO_2_ NPs, which is accompanied by increased expression of mitochondrial apoptotic proteins including Bax, Caspase-3, Caspase-9, and Cytochrome C (Cyt C) and increased expression of autophagy-related proteins including Atg5, Atg12, Beclin-1, and LC3-II. Treating HeLa cells with N-acetyl-L-cysteine (NAC) significantly reduced ROS, rate of apoptosis, MMP, and *in vivo* anti-tumor activity. In addition, apoptosis- and autophagy-related protein expressions were also suppressed. Based on these observations, we conclude that ZrO_2_ NPs induce HeLa cell death through ROS mediated mitochondrial apoptosis and autophagy.

## Introduction

ZrO_2_ NPs are the main raw material for synthetic casting sand, refractory and porcelain. In addition, they are common biomedical materials used for biosensors, implants, joints and oral prosthesis replacement (Lohbauer, et al., 2010; Qeblawi, et al., 2010). However, their anti-tumor activity is rarely reported. Reduced graphene oxide modified by ZrO_2_ NPs are used for highly sensitive electrochemical sensors for anticancer drugs (Venu, et al., 2018). Iron-manganese-doped sulfated zirconia nanoparticles have been shown to induce HepG2 and MDA-MB-231 cell death, while they are less effective at killing normal Chang cells and HUVECs (Al-Fahdawi, et al., 2015). Sulphated zirconia nanoparticles have significant cytotoxic effects on colon cancer HT29 cells (Mftah, et al., 2015). Synthetic ZrO_2_ NPs can play a role in biomedical applications through the production of ROS and antioxidant activities (Balaji, et al., 2017), but the detailed mechanism has not been explored. Based on this, we investigated the anti-tumor activity and anti-tumor mechanism of zirconia nanoparticles using *in vivo* and *in vitro* experiments.

ROS are molecules or ions composed of oxygen containing single unpaired electrons, including hydroxyl radicals, superoxides, and hydrogen peroxide, etc (Chen and Gibson, 2008). They play important roles in cell apoptosis and autophagy (Araveti and Srivastava, 2019; Wang, et al., 2019; Ye et al., 2019). Mitochondria are the main organelles for ROS production (Addabbo, et al., 2009). Although autophagy is the protective response of living organisms to various adverse stress conditions during evolution, excessive autophagy is another way for anti-tumor drugs to induce tumor cell death (Kroemer and Levine, 2008). Overproduction of ROS can promote autophagy (Chen and Gibson, 2008). Based on the close relationship between ROS and apoptosis and autophagy, in this study, we investigated the anti-tumor mechanism of ZrO_2_ NPs focusing ROS generation, apoptosis and autophagy induction.

## Materials and Methods

### Materials

Zirconia nanoparticle (XF101) was purchased from XFNANO Materials Tech Co., Ltd. (China). NAC was purchased from Sigma Aldrich (United States). MTT cell proliferation and cytotoxicity assay kit was purchased from Beyotime Biotechnology. Rhodamine 123 (Rh123) was obtained from Yeasen Biotechnology (China). Annexin V-FITC/PI Apoptosis Detection Kit, MMP Detection Kit (JC-1), and ROS Assay Kit were purchased from KeyGEN BioTECH (China). BCA Protein Assay Kit was purchased from Solarbio science and technology Co., Ltd. (China). DMEM-HG (high glucose), fetal bovine serum (FBS), and trypsin were purchased from Life Technologies (United States). BBoxiProbe^®^ superoxide anion detection kit, Singlet oxygen detection kit were from Shanghai Bestbio Biotechnology Co., Ltd. (China). Hydroxyl radical *in situ* fluorescence staining kit was from Shanghai Haling Biological Technology Co., Ltd. (China). One Step TUNEL Apoptosis Assay Kit, Ki-67 cell proliferation Detection Kit (IHC), DAPI and hematoxylin and eosin (HE) were purchased from Servicebio Tech Co., Ltd. (China). Anti-Bax, anti-Bcl-2, anti-Caspase-9, anti-Caspase-3, anti-Cytochrome C, anti-LC3, anti-Atg12, anti-Atg5, anti-Beclin-1, anti-β-actin antibodies, and horseradish peroxidase (HRP) goat anti-mouse and goat anti-rabbit IgG secondary antibodies were produced by Proteintech (United States).

### Cell Culture

Human cervical cancer HeLa cells were prepared in the laboratory and cultured with DMEM-HG, containing 10% fetal bovine serum at 37°C, in 5% CO_2_ and saturated humidity condition.

### Cell Viability and Mitochondrial Metabolic Activity Detected by MTT Cell Proliferation and Cytotoxicity Assay

HeLa cells growing in log phase were collected and inoculated in 96-well plate (2 × 10^3^/well). After 24 h, confluent cells were divided into control group, NAC (160 μg/ml) group, ZrO_2_ NPs (100 μg/ml) + NAC (160 μg/ml) group, ZrO_2_ NPs (50 μg/ml) group, and ZrO_2_ NPs (100 μg/ml) group, respectively. After 24 h, 10 μL MTT solution was added to each well and the plate was incubated for 4 h. 100 μl Formazan solution was added to each well and the plate was incubated for additional 4 h. The absorbance (A) at 570 nm was detected by an EnSpire 2300 Multilabel Plate Reader. Cell viability (%) = (1 − average A value of ZrO2 NPs group/average A value of negative control group) × 100%. The experiment was performed in triplicates.

### Preparation of Samples for TEM

After being treated with fresh medium, ZrO_2_ NPs (100 μg/ml) + NAC (160 μg/ml), ZrO_2_ NPs (50 μg/ml), or ZrO_2_ NPs (100 μg/ml) for 24 h, respectively, HeLa cells were collected by trypsin digestion, washed with PBS and centrifuged at 1,000 rpm for 5 min. PBS wash was repeated three times. Cell clumps (about 1–2 mm^3^) were fixed in 3% glutaraldehyde at 4°C for 2 h, after being washed with PBS, and then fixed with 1% osmic acid for 2 h. After dehydration with ethanol and acetone, cells were embedded with Epon821 and followed by polymerization. Cell clumps were sliced by an ultra-thin slicing machine, counterstained with oil and citric lead acetate, and then observed under TEM (GEM-100-CX Ⅱ, JEO, Japan) and pictures were taken.

### Apoptosis Assay by Annexin V-FITC/PI Double Staining

HeLa cells were inoculated into culture bottles, treated with fresh medium, ZrO_2_ NPs (100 μg/ml) + NAC (160 μg/ml), ZrO_2_ NPs (50 μg/ml), or ZrO_2_ NPs (100 μg/ml), respectively. After 24 h, cells were digested with Trypsin that did not contain EDTA, collected, washed with pre-cooled PBS twice, and centrifuged (2000 rpm, 5 min) at 4°C. 1–5 × 10^5^ cells were collected. PBS was discarded, and 100 μl 1x Binding Buffer was added to re-suspend the cells. 5 μl Annexin V-FITC and 10 μl PI Staining Solution was added and mixed gently. The cells were incubated in dark at room temperature for 10–15 min. 400 μl 1x Binding Buffer was added, and the cells were suspended. The samples were subjected to FCM (BectonDickinson-LSR, United States) within 1 h.

### MMP Assay

HeLa cells were inoculated into culture bottles, treated with fresh medium, ZrO_2_ NPs (100 μg/ml) + NAC (160 μg/ml), ZrO_2_ NPs (50 μg/ml), or ZrO_2_ NPs (100 μg/ml), respectively. After 24 h, cells were collected, washed, and suspended with 500 μl PBS. 500 μl Rhodamine 123 (10 mg/L) was added to each cell suspension. After incubation at 37°C for 30 min, cells were washed with PBS three times, and suspended with PBS. FCM (BectonDickinson-LSR, United States) was used to detect the MMP of HeLa cells. The excitation wavelength is 488 nm and the emission wavelength is 525 nm.

### Reactive Oxygen Species Detection

HeLa cells were inoculated into culture bottles, treated with fresh medium, ZrO_2_ NPs (100 μg/ml) + NAC (160 μg/ml), ZrO_2_ NPs (50 μg/ml), or ZrO_2_ NPs (100 μg/ml), respectively. Cells were collected after 24 h and washed with PBS three times. The cells were collected and suspended in DCFH-DA (10 mmol/L) with a cell concentration of 1–20 × 10^6^/ml, and incubated at 37°C for 20 min. The cells were washed three times with serum-free cell culture medium to fully remove DCFH-DA, and cellular ROS level was detected by FCM (BectonDickinson-LSR, United States).

### Superoxide Anion, Singlet Oxygen, and Hydroxyl Radical Detection

To further explore which kinds of ROS were generated by ZrO_2_ NPs, we detected the superoxide anion, singlet oxygen, and hydroxyl radicals in HeLa cells after treated with ZrO_2_ NPs.

#### Superoxide Anion in HeLa Cells Detected by Superoxide Anion Detection Kit After Treated With ZrO_2_ NPs

The 100 fold diluted probe was added to serum-free cell culture medium, and the cells were incubated at 37°C for 2 h in dark, washed twice with PBS, observed and photographed under fluorescence microscope (ECLIPSE TE2000-U, Nikon, Japan, excitation wavelength: 518 nm, emission wavelength: 606 nm).

#### Singlet Oxygen in HeLa Cells Detected by Singlet Oxygen Detection Kit After Treatment With ZrO_2_ NPs

The 100 fold diluted singlet oxygen R probe was added to serum-free cell culture medium, and the cells were incubated at 37°C for 2 h in the dark, washed twice with PBS, and observed and photographed under fluorescence microscope (ECLIPSE TE2000-U, Nikon, Japan, excitation wavelength: 488 nm, emission wavelength: 526 nm).

#### Hydroxyl Radicals in HeLa Cells Were Detected by Hydroxyl Radical *In Situ* Fluorescence Staining Kit After Treated With ZrO_2_ NPs

The cell medium was discarded and 500 μl of Reagent A added. Cleaning agent (Reagent A) was disposed of, then 500 μl of Reagent B and Reagent C were added, then incubated with cells at 37°C in cell incubator for 30 min. The staining agent was discarded, and 500 μl of preheated Reagent D was added at 37°C and observed and photographed with inverted fluorescence microscope (ECLIPSE TE2000-U, Nikon, Japan, excitation wavelength: 499 nm, emission wavelength: 515 nm).

### Establishment of Xenograft Animal Model

Female BALB/c nude mice aged 6 weeks (about 20 g) were purchased from Hunan Slake Jingda Laboratory animal Co. Ltd. Xenograft tumor models of nude mice were prepared by subcutaneous inoculation of HeLa cells at 1 × 10^7^/100 μl.

### Anticancer Effect of ZrO_2_ NPs on Nude Mice With Transplanted Tumor

Day 1 was defined when tumor volume reaches 100 mm^3^. Female BALB/c nude mice were randomly divided into four groups with five in each group. PBS, ZrO_2_ NPs (50 mg/Kg/d) + NAC (80 mg/Kg/d), ZrO2 NPs (25 mg/Kg/d), and ZrO2 NPs (50 mg/Kg/d) was injected into the tail vein in 100 μl daily for four consecutive days. Tumor volume and body weight were measured every 4 days. On the 20^th^ day, mice were anesthetized and sacrificed. Whole blood, tumor, and tissues (heart, liver, spleen, lung, and kidney) were collected. Whole blood was collected with EDTA anticoagulation, and analyzed on BC-5390 (Mindray, China). Whole blood samples were centrifuged at 3,000 rpm for 10 min, and the serum enzymatic indexes were detected by automatic biochemical analyzer (7100, HITACHI, Japan) and immune analyzer (Cobas 6000 e601, ROCHE, United States). All the organs and tumor tissues were either fixed with 4% paraformaldehyde or stored in −80°C until use. Frozen tissue sections were prepared for immunofluorescence staining and Western Blotting. Fixed tissues were embedded in paraffin and made into tissue sections for HE staining, immunofluorescence and immunohistochemical staining.

### Immunohistochemistry and Immunofluorescence Staining

#### Ki-67 Assay

Paraffin-embedded tumor sections were dewaxed and antigen repaired in the antigen repair solution. Slices were heated to 95°C for 20 min, then slowly cooled to 65°C, and placed in the elution buffer for 5 min. The slices were incubated with peroxidase blocker, rinsed with elution buffer for 5 min, incubated with primary antibody for 20 min, washed with elution buffer, incubated with secondary antibody for 20 min, and flushed with elution buffer. Then DAB was applied for color rendering. The slices were rinsed with tap water, stained with hematoxylin, and then rinsed in 70% alcohol, 80% alcohol, 90% alcohol, 100% alcohol, and 100% alcohol for 2 min, respectively. The slices were vitrified by dimethylbenzene. The slices were sealed with neutral gum, observed and photographed under a light microscope.

#### TUNEL, MMP and ROS Assay

Frozen sections of tumor tissues were used to detect the apoptosis according to the instructions of One Step TUNEL Apoptosis Assay Kit. The cells were fixed for 30–60 min with 4% paraformaldehyde. The slices were washed with PBS twice for 10 min each time, incubated in PBS containing 0.5% Triton X-100 at room temperature for 5 min, washed twice with PBS, incubated with 50 μl TUNEL test solution at 37°C in the dark for 60 min, and washed with PBS three times. The nuclei were counterstained with DAPI. The slices were sealed with anti-fluorescence quenching solution.

Frozen sections of tumor tissue were incubated with JC-1 staining solution at 37°C for 20 min, washed with JC-1 staining buffer twice. The nuclei were counterstained with DAPI.

Frozen tissue slices were incubated in DCFH-DA (10 mmol/L) at 37°C for 20 min and washed three times with PBS to fully remove DCFH-DA. The nuclei were counterstained with DAPI. All the tissue sections were observed and photographed under fluorescence microscope (ECLIPSE TE2000-U, Nikon, Japan).

### Western Blot Detection of Apoptosis and Autophagy-Related Proteins

The cryopreserved tumor tissues were blended with cell lysis solution for 40 min, centrifugated (13,000 rpm, 20 min) at 4°C, and supernatant was taken for protein quantification with BCA Protein Assay Kit. Protein samples were separated by 80 V electrophoresis and transferred to nitrocellulose membrane. Sealed membrane protein was blotted with Tris Buffered Saline Tween (TBST) buffer containing 5% skim milk at room temperature for 1 h, and then blotted at room temperature for 1 h with anti-Bax, anti-Bcl-2, anti-Caspase-3, anti-Caspase-9, anti-Cytochrome C, anti-Beclin-1, anti-LC3, anti-Atg5, anti-Atg12, and anti-β-actin antibody, respectively. Membranes were washed with TBST three times, 10 min each, and then incubated with horseradish peroxidase (HRP)-labeled goat anti-mouse or goat anti-rabbit IgG secondary antibody at room temperature for 1 h. Membranes were washed with TBST three times, 10 min each, reacted with enhanced chemical illuminant (ECL), and developed by exposure on X film.

### Image and Statistical Analysis

SPSS 20.0 software was used for statistical analysis, and GraphPad Prism plot was used to plot the data. Data was expressed as mean ± standard deviation. ANOVA was used to evaluate the differences between groups, and Tukey's post-test was conducted. ^*^ *p* < 0.05, ^**^ *p* < 0.01, ^***^ *p* < 0.001, and ^****^ *p* < 0.0001.

## Results and Discussion

### Characterization of Zirconia Nanoparticles

**FIGURE 1:**
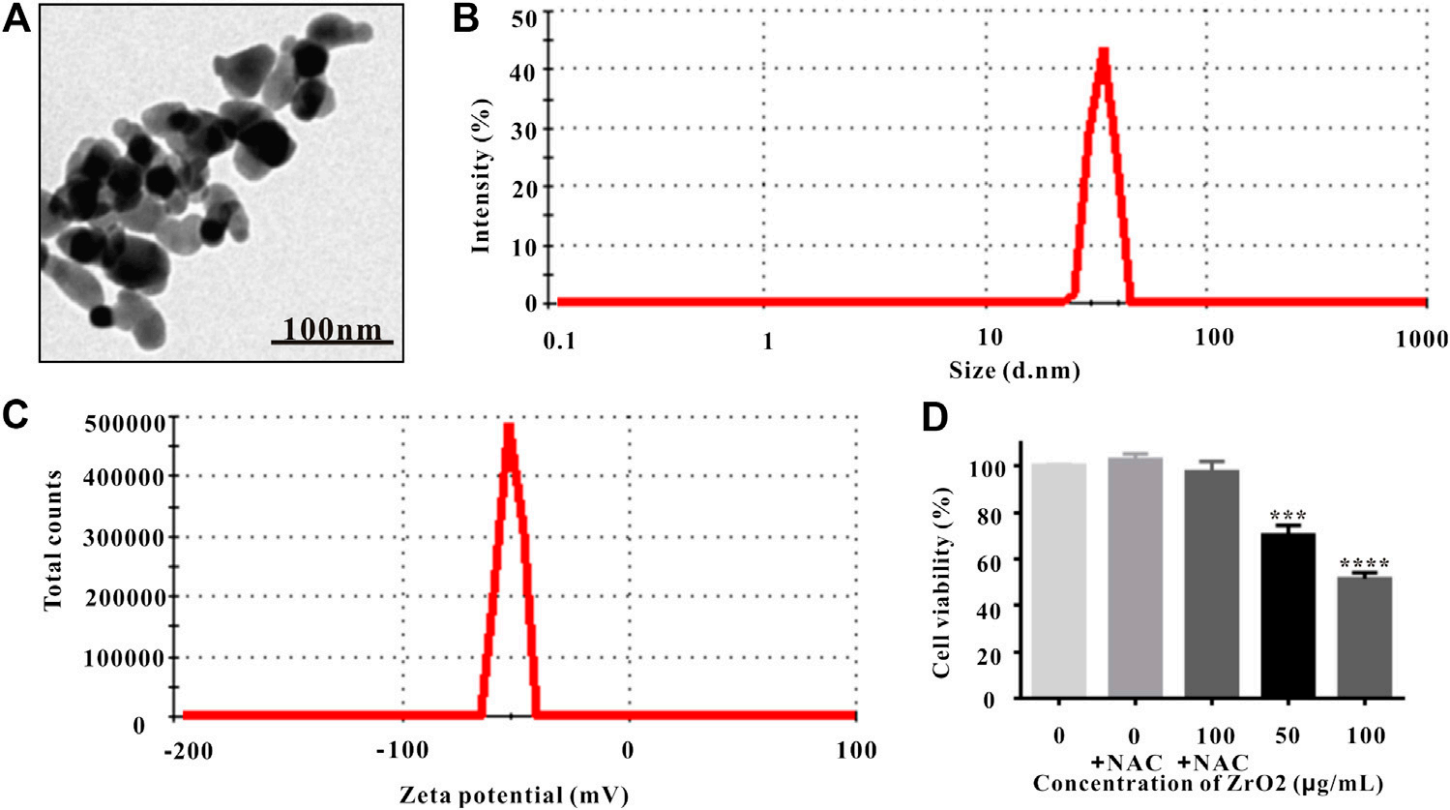
Characterization of ZrO_2_ NPs. **(A)** TEM image of ZrO_2_ NPs. Scale bar: 100 nm. **(B)** The particle size of ZrO_2_ NPs. **(C)** Zeta potential of ZrO_2_ NPs. **(D)** HeLa cell viability upon administration of NAC, ZrO_2_ NPs, and ZrO_2_ NPs + NAC for 24 h, respectively. Data are mean ± SD (*n* = 3). Compared to the control group: ***p < 0.001, and ****p < 0.0001.

Observed under TEM, ZrO_2_ NPs were monodispersed with diameters averaging about 25 nm (Figure 1A), which were larger than the previous reported green synthesized Nanozirconia (of ∼9–11 nm) using leaf extract of *Eucalyptus globulus* (*E. globulus*) (Balaji, et al., 2017). Based on dynamic light scattering (DLS) data (Figure 1B), ZrO_2_ NPs averaged 25 nm in size, which was consistent with the data of TEM. Zeta potential is related to colloid stability of nanoparticle-dispersions, and Zeta potential values of ±0–10, ±10–20, and ±20–30 mV and >±30 mV are classified as highly unstable, relatively stable, moderately stable and highly stable (Bhattacharjee, 2016). Nanoparticles with low Zeta potential are easy to agglomerate (Hanaor et al., 2012). ZrO_2_ NPs had Zeta potential values of −51.5 ± 3.1 mV (Figure 1C), which was higher than the reported green synthesized Nano zirconia (−45.5 mV) (Balaji et al., 2017). Besides, Zeta potentials of ZrO_2_ NPs over 180 days did not change significantly (Supplementary Figure S1). Taking all these into account, ZrO_2_ NPs exhibit higher colloid stability and anti-agglomeration tendency. The X-Ray Diffraction (XRD) pattern indicates that the fitting line is relatively flat and the fitting is very good (Supplementary Figure S2A), and the crystallinity of ZrO_2_ NPs is 99.3% (Supplementary Figure S2B).

### Cell Viability and Mitochondrial Metabolic Activity of HeLa Cell Growth

MTT assay is widely used to assess cell viability, and the involvement of mitochondrial metabolic activity in MTT reduction has been confirmed (Berridge and Tan, 1993). NAC, the ROS scavenger, was utilized to suppress ROS accumulation (Singh, et al., 2005; Kim, et al., 2006). MTT assay showed that NAC did not inhibit the viability and mitochondrial metabolic activity of HeLa cells, but the cell viability and mitochondrial metabolic activity of HeLa cells was significantly inhibited (Figure 1D). Treating with ZrO_2_ NPs together with NAC did not show obvious inhibitory effect (Figure 1D), indicating NAC could counter the cytotoxicity induced by ZrO_2_ NPs and ZrO_2_ NPs could inhibit the growth of HeLa cells through ROS production.

### Ultrastructure Changes of HeLa Cells Observed Under TEM

**FIGURE 2:**
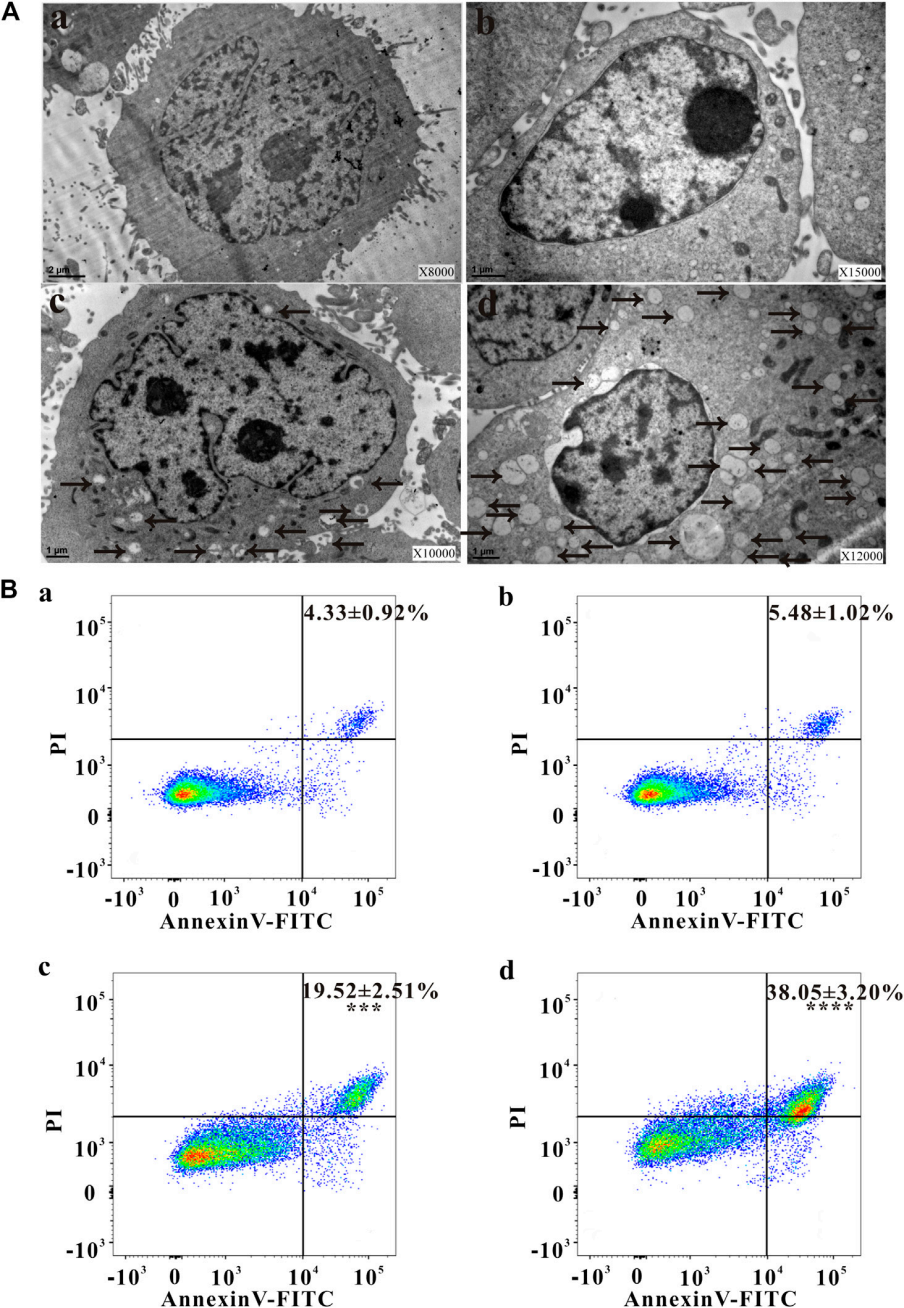
Changes of ultrastructure, apoptosis ratio, MMP and ROS level of HeLa cells. **(A)** The ultrastructural changes of HeLa cells after treatment with ZrO_2_ NPs, and ZrO_2_ NPs + NAC for 24 h, respectively. **(B)** Apoptosis ratio of HeLa cells after treatement with ZrO_2_ NPs and ZrO_2_ NPs + NAC detected by Flow Cytometer. a: Control; b: ZrO_2_ NPs (100 μg/ml) + NAC (160 μg/ml); c: ZrO_2_ NPs (50 μg/ml); d: ZrO_2_ NPs (100 μg/ml). Data are presented as the mean ± SD (n = 3). Compared to the control group: ***p < 0.001 and ****p < 0.0001.

Autophagy has been involved in pathological conditions such as cancer and inflammatory diseases (Choi, et al., 2013). Promotion of autophagy has been a potent therapeutic target in human diseases, including tumor (Yoshii and Mizushima, 2017). As shown in Figures 2Aa,Ab, no autophagic vacuoles were found in the cytoplasm of HeLa cells, but lots of autophagosomes (black arrows) were present in the apoptotic cells (Figures 2Ac,Ad), indicating NAC inhibited the autophagy induced by ZrO_2_ NPs. Apoptotic cell death is an important mechanism of anti - tumor therapy and most anticancer drugs exploit apoptotic signaling pathways to induce cancer cell death (Pistritto et al., 2016). TEM images also showed that autophagy was associated with apoptosis. The membrane of HeLa cell in the control group was intact, while the nuclei, nuclear membrane and nucleoli were clearly visible (Figure 2Aa). After ZrO_2_ NPs treatment, cells appeared wrinkled while the chromatin became dense and gathered under the nuclear membrane. The mitochondria were swollen and expanded, but the cell membrane was still intact indicating apoptosis (Figures 2Ac,Ad). After ZrO_2_ NPs plus NAC treatment, the swelling and enlargement of mitochondria in the cells was not obvious, suggesting that NAC inhibited the apoptosis induced by ZrO_2_ NPs (Figure 2Ab).

### Cell Apoptosis Rate, MMP Changes, and ROS Levels Detected by FCM

Escaping from apoptosis is a hallmark of cancer and promoting apoptosis is an interesting therapeutic strategy (Pistritto, et al., 2016). The percentage of apoptotic cells in the control group was low (4.33 ± 0.92%), but apoptosis increased after cells were treated with ZrO_2_ NPs (100 μg/ml), reaching 38.05 ± 3.20%. After ZrO_2_ NPs plus NAC treatment, the percentage of apoptotic cells was significantly lower than that in the ZrO_2_ NPs group, showing that NAC inhibited apoptosis induced by ZrO_2_ NPs (Figure 2B).

**FIGURE 3:**
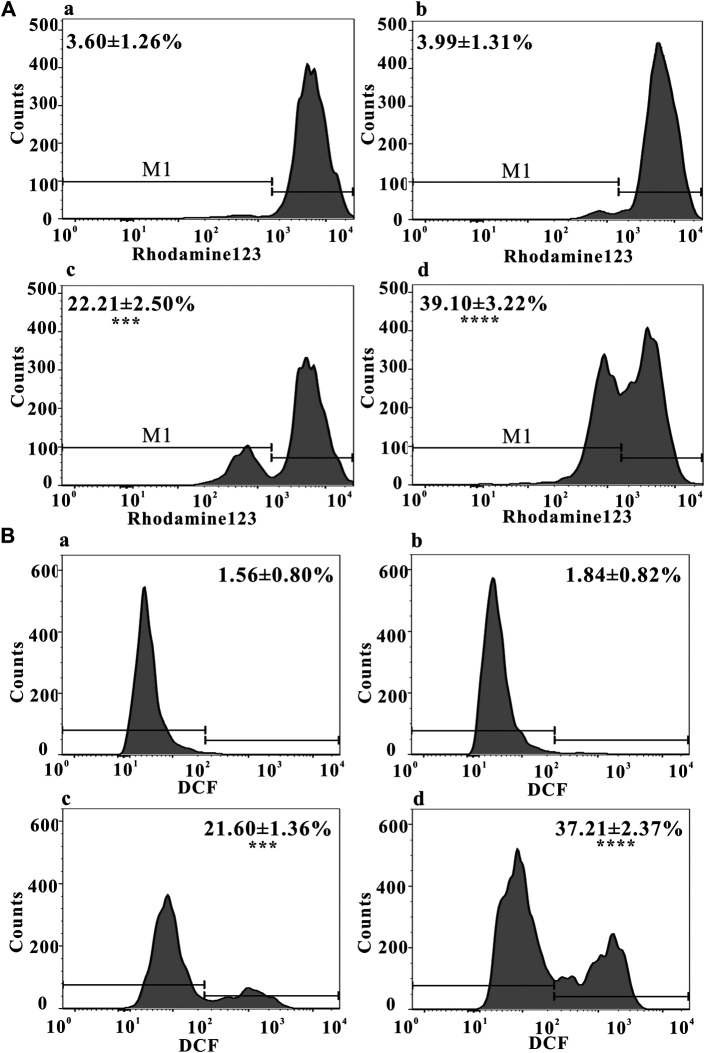
**(A)** MMP of HeLa cells after treatment with ZrO_2_ NPs and ZrO_2_ NPs + NAC detected by Flow Cytometer. **(B)** ROS level of HeLa cells after treatment with ZrO_2_ NPs and ZrO_2_ NPs + NAC detected by Flow Cytometer. a: Control; b: ZrO_2_ NPs (100 μg/ml) + NAC (160 μg/ml); c: ZrO_2_ NPs (50 μg/ml); d: ZrO_2_ NPs (100 μg/ml). Data are presented as the mean ± SD (*n* = 3). Compared to the control group: ***p < 0.001 and ****p < 0.0001.

Mitochondrial function, a key indicator of cell apoptosis, can be assessed by monitoring changes in MMP (Sakamuru, et al., 2016). MMP reflects the functional status of the mitochondrion (Zhang, et al., 2015a), and a decrease in MMP is linked to apoptosis (Lemasters, et al., 2002). As shown in Figure 3A, after treating with ZrO_2_ NPs + NAC for 24 h, the proportion of HeLa cells with weak fluorescence (M1 channel) was 3.99 ± 1.31%, which was close to that in the control group (3.60 ± 1.26%). The proportion of cells with weak fluorescence (M1 channel) in the ZrO_2_ NPs (100 μg/ml) groups increased significantly, reaching 39.10 ± 3.22%. This result suggested that ZrO_2_ NPs decreased the binding ability of mitochondria to Rhodamine 123, resulting in a decrease of fluorescent dyes entering cells and an increase of the percentage of cells with weak fluorescence, and a decrease of MMP, while NAC could significantly inhibit this effect.

Mitochondria and potentially mitochondrial ROS play an important role in regulating apoptosis (Bender and Martinou, 2013) and autophagy (Chen et al., 2009). As shown in Figure 3B, the proportion of DCF-positive cells was 1.56 ± 0.80% in the control group, 1.84 ± 0.82% in the ZrO_2_ NPs + NAC group, 21.60 ± 1.36% and 37.21 ± 2.37% in the ZrO_2_ NPs 50 and 100μg/ml groups, respectively, suggesting that ZrO_2_ NPs promote ROS production in HeLa cells, while NAC significantly inhibits ROS production induced by ZrO_2_ NPs. ROS generation by ZrO_2_ NPs is likely caused a decrease in *n* →π* transition intensity due to the transfer of electron from oxygen atom present in ZrO_2_ NPs (Balaji, et al., 2017). Further, ZrO_2_ NPs may inhibit the scavenging of free radicals (Balaji, et al., 2017).

### Generation of Superoxide Anion, Singlet Oxygen, and Hydroxyl Radicals After Treated With Zirconia Nanoparticles

#### Superoxide Anion Assay

The Bestbio ^®^ BBoxiProbe^®^ superoxide anion detection kit is a superoxide anion detection kit that utilizes the BBoxiProbe^®^ O88 superoxide anion specific fluorescent probe. BBoxiProbe ^®^ O88 can enter the cell freely through the living cell membrane and is oxidized by the intracellular superoxide anion to produce red fluorescence products. According to the production of red fluorescence in living cells, the amount and change of cell superoxide anion content can be determined. Superoxide anion was transformed from an oxygen molecule which accepted an electron. Repeated such reactions lead to an excess of superoxide anions, resulting in cytotoxicity (Sun, et al., 2020). After treatment with ZrO_2_ NPs, red fluorescence in HeLa cells enhanced (Supplementary Figure S3A), indicating generation of superoxide anion to induce cell death.

#### Singlet Oxygen Assay

Singlet oxygen specific fluorescent probe *R* in Singlet oxygen detection kit was used to detect singlet oxygen. Singlet oxygen probe *R* is a synthetic phenylanthracene fluorescent probe, which can freely enter cells, react with singlet oxygen in cells, and be oxidized to produce green fluorescent substances. The intensity of green fluorescence is proportional to the level of singlet oxygen in cells, and the changes of singlet oxygen in cells can be known by detecting green fluorescence. O_2_ derivatives (such as singlet oxygen and hydroxyl radical), owing to their redox potential, can promote cell death (Bubici, et al., 2006). After being treated with ZrO_2_ NPs, green fluorescence in HeLa cells was enhanced (Supplementary Figure S3B), indicating that ZrO_2_ NPs increased the generation of singlet oxygen to induce HeLa cells death.

#### Hydroxyl Radical Assay

Hydroxyphenyl fluorescein (HPF) is a kind of dye that passes through cell membrane freely. Once it reacts with hydroxyl radicals, it generates o-dearylation and fluorescein. These results proved the existence of hydroxyl radical reactive oxygen group in cells. Enhanced green fluorescence indicates high hydroxyl radical content. Excess hydroxyl radical (OH) can promote oxidation and stimulate lipid peroxidation (Polyakov, et al., 2018), damage DNA and protein, and promote the apoptosis of cancer cells (Lin et al., 2018). After being treated with ZrO_2_ NPs, green fluorescence in HeLa cells was enhanced (Supplementary Figure S3C), indicating that ZrO_2_ NPs can promote the generation of hydroxyl radical, which may play an important role in inducing HeLa cells apoptosis.

### Antitumor Effect of Zirconia Nanoparticles *in vivo*

**FIGURE 4:**
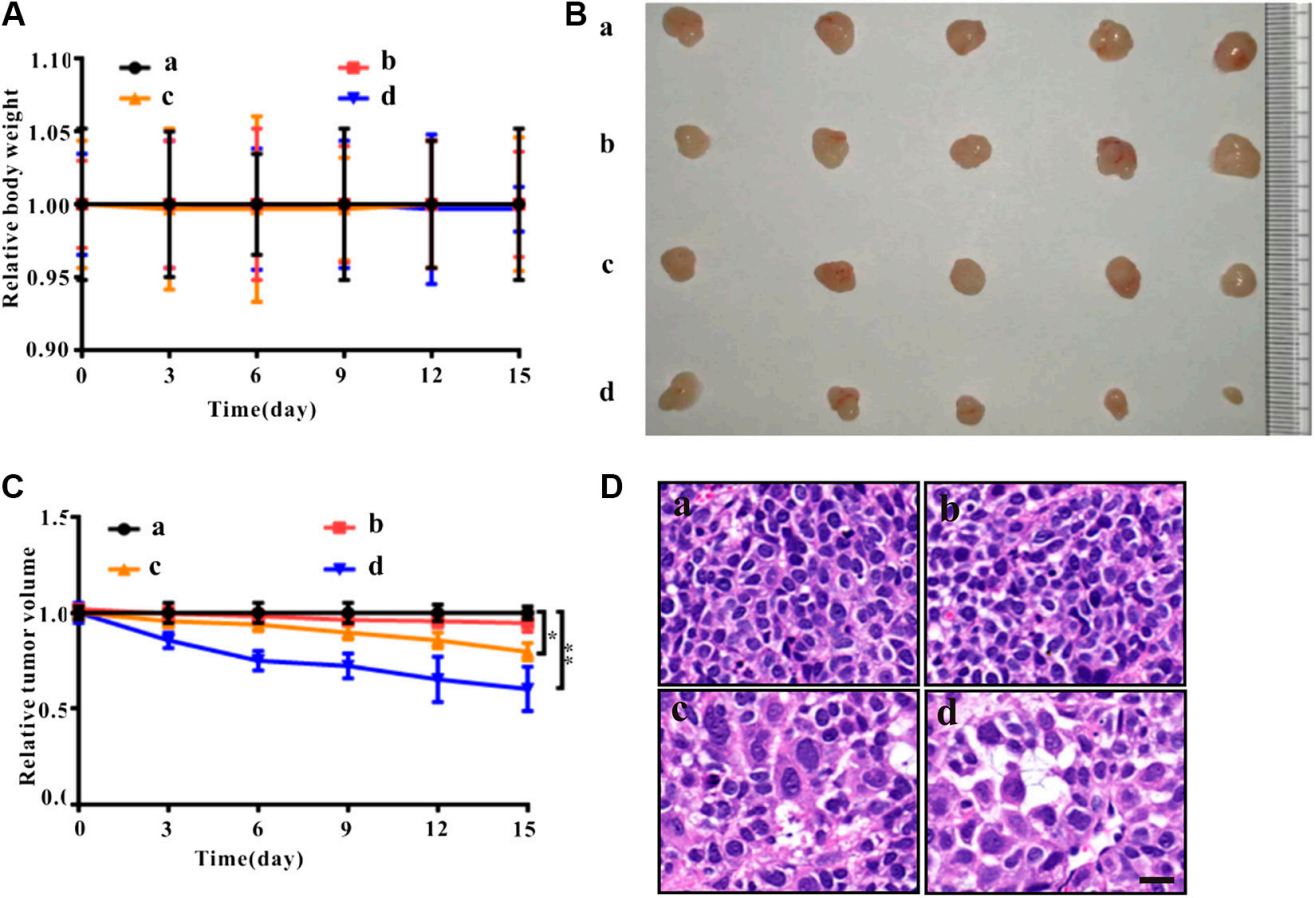
Anticancer effects of ZrO_2_ NPs and ZrO_2_ NPs + NAC on HeLa bearing BALB/c nude mice. **(A)** The change of body weight after treated with ZrO_2_ NPs and ZrO_2_ NPs + NAC. **(B)** The representative picture of tumors. **(C)** The change of tumor volume after treated with ZrO_2_ NPs and ZrO_2_ NPs + NAC. **(D)** The representative cell morphology of tumor tissues after treated with ZrO_2_ NPs and ZrO_2_ NPs + NAC, and HE staining. Scale bar: 20 μm. a: Control; b: ZrO_2_ NPs (50 mg/Kg/d) + NAC (80 mg/Kg/d); c: ZrO_2_ NPs (25 mg/Kg/d); d: ZrO_2_ NPs (50 mg/Kg/d). Data are presented as the mean ± SD (*n* = 3). Compared to the control group: ***p < 0.001 and ****p < 0.0001.

As shown in Figure 4A, compared to the control group, there was no significant change in body weight of nude mice treated with ZrO_2_ NPs and ZrO_2_ NPs + NAC. As shown in Figures 4B,C, compared to the control group, tumor volume decreased after ZrO_2_ NPs treatment, suggesting that ZrO_2_ NPs inhibit tumor growth. However, ZrO_2_ NPs plus NAC treatment did not further decrease tumor volume. After HE staining, tumor tissue sections (Figure 4D) showed tumor cells were thriving in the control group and the ZrO_2_ NPs + NAC group. After ZrO_2_ NPs treatment, necrotic HeLa cells increased, suggesting that ZrO_2_ NPs inhibit HeLa cell growth while NAC counteracts the inhibition effect induced by ZrO_2_ NPs.

### Immunohistochemistry and Immunofluorescence Staining of Tumor Sections

#### Ki-67 Immunohistochemistry and TUNEL Immunofluorescence Assays

**FIGURE 5:**
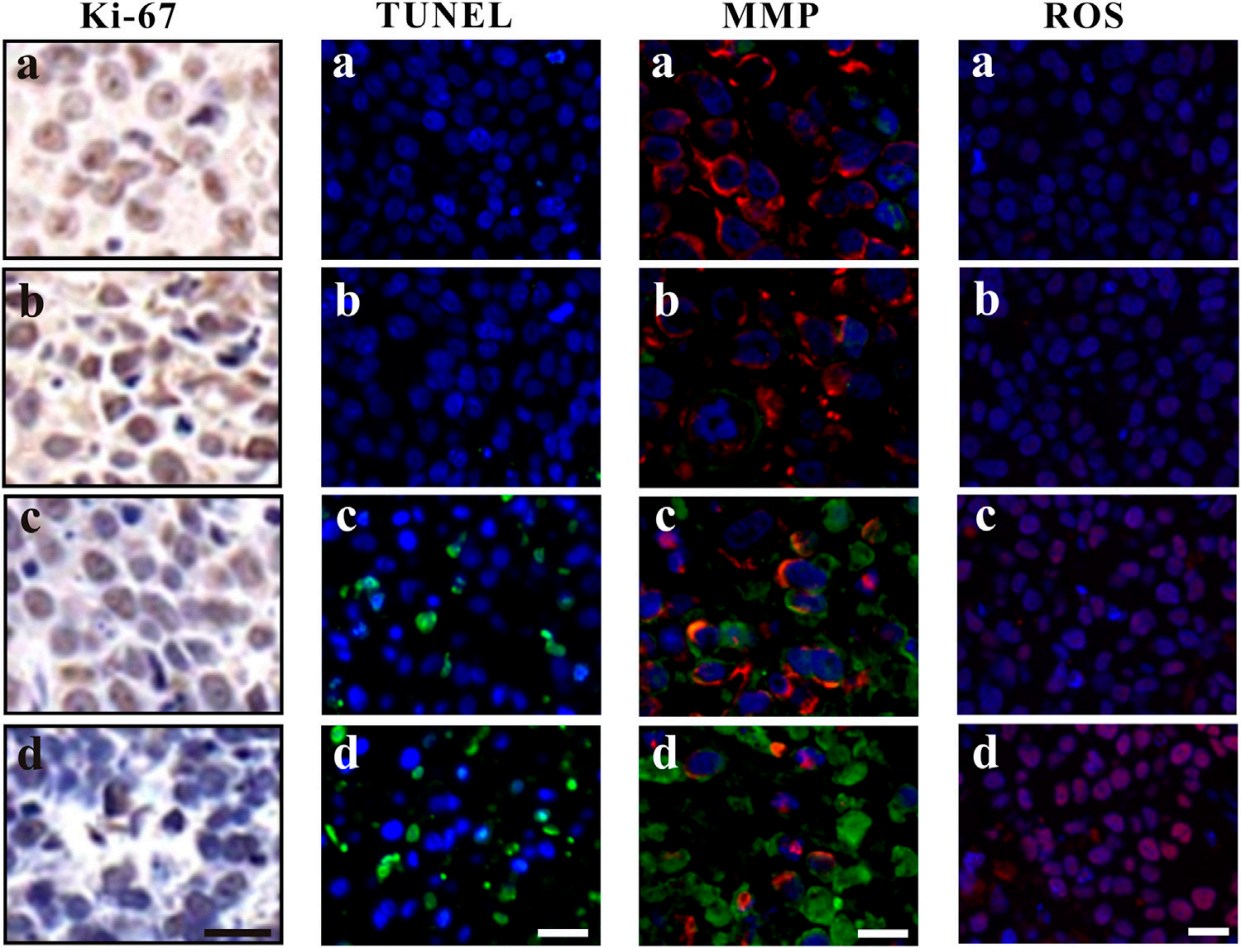
Ki-67 assay, TUNEL assay, MMP assessment and ROS analysis of tumor tissues at 20th day after the intravenous injection of ZrO_2_ NPs and ZrO_2_ NPs + NAC. a: Control; b: ZrO_2_ NPs (50 mg/Kg/d) + NAC (80 mg/Kg/d); c: ZrO_2_ NPs (25 mg/Kg/d); d: ZrO_2_ NPs (50 mg/Kg/d). Scale bar: 20 μm.

Ki-67, a nuclear and nucleolar protein, is associated with cell proliferation (Campelo, et al., 2015). As shown in Figure 5, compared to the control group, Ki-67-positive (brown) cells decreased after ZrO_2_ NPs treatment, while the decrease of Ki-67 labeling was not apparent in the ZrO_2_ NPs + NAC group suggesting that ZrO_2_ NPs could inhibit HeLa cell proliferation, and NAC could counter the inhibitory effect induced by ZrO_2_ NPs. TUNEL assay has been designed to detect apoptotic cells that undergo extensive DNA degradation during the late stages of apoptosis, which is based on the ability of TdT to label blunt ends of double-stranded DNA breaks independent of a template (Kyrylkova, et al., 2012). As shown in Figure 5, compared to the control group, TUNEL-positive (green fluorescent) cells increased after ZrO_2_ NPs treatment, but no obvious change was detected in the ZrO_2_ NPs + NAC group, suggesting that ZrO_2_ NPs induce apoptosis of HeLa cells and that NAC reverses the apoptosis induced by ZrO_2_ NPs.

#### MMP and ROS Immunofluorescence Staining

The decrease of MMP is the direct consequence of the imbalance between pro-apoptosis factor and anti-apoptosis factor, which leads to altered mitochondrial permeability and triggers an early and subsequent intracellular event in apoptosis (Cetindere, et al., 2010; Indran, et al., 2011). The lipophilic cation JC-1 has been used as a specific dye for measuring MMP (De Biasi, et al., 2015). As shown in Figure 5, cells of tumor tissue sections in the control group and ZrO_2_ NPs + NAC group showed robust staining with JC-1, indicating active proliferation. However, cells in the ZrO_2_ NPs group had a significant decrease of MMP as indicated by the increase of green fluorescent cells, suggesting that ZrO_2_ NPs result in MMP reduction in HeLa cells. ROS formation has long been found to play a vital role in mediating apoptosis (Pierce, et al., 1991). As shown in Figure 5, DCFH-DA intensity was much stronger in tumor cells treated with ZrO_2_ NPs than that of the control group and ZrO_2_ NPs + NAC group, and the intensity increases as the dose of ZrO_2_ NPs increases, suggesting that ZrO_2_ NPs induce tumor cells to produce ROS while NAC could inhibit the ROS production induced by ZrO_2_ NPs.

### Changes in Apoptosis and Autophagy-Associated Proteins Expression

**FIGURE 6:**
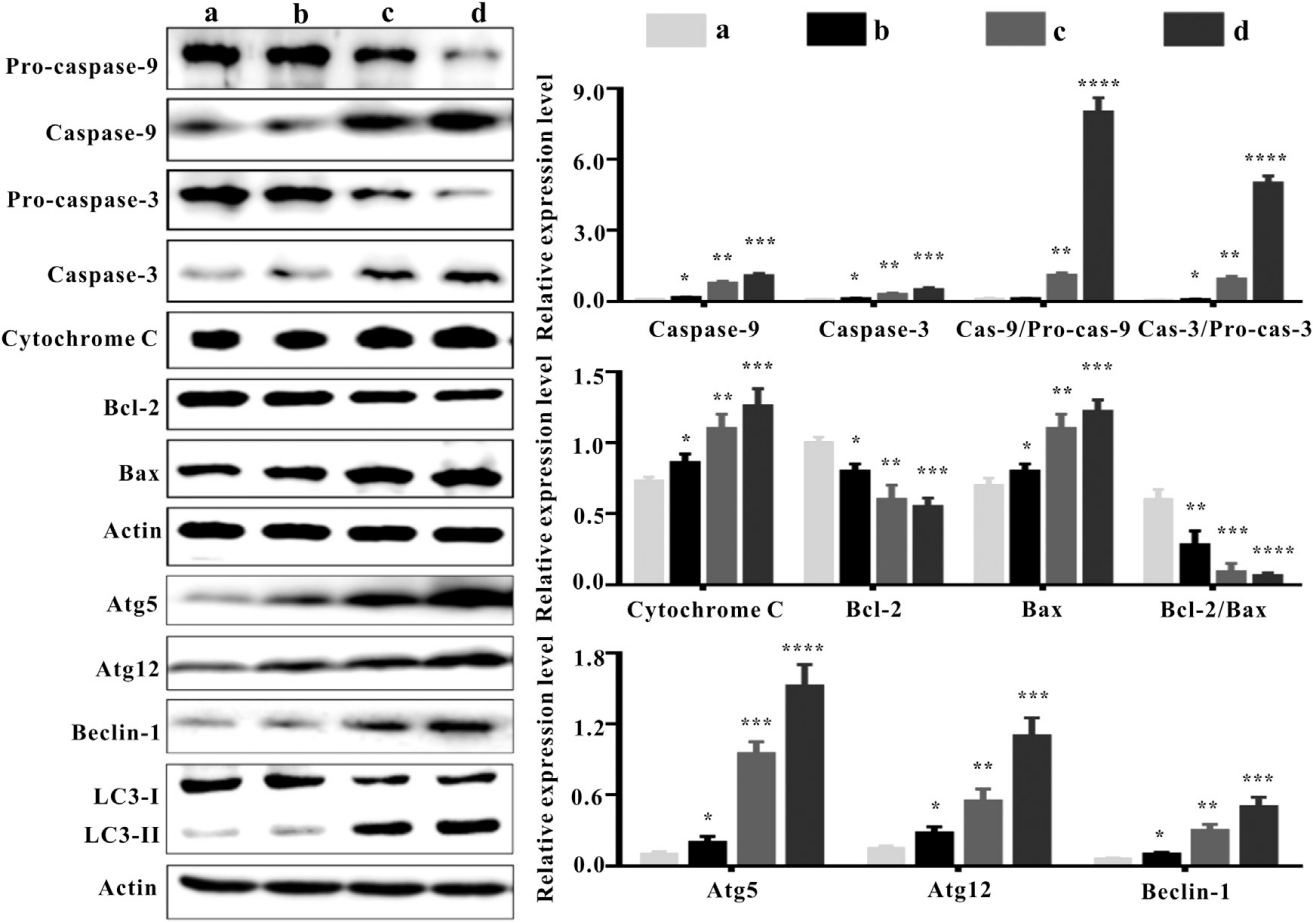
Apoptosis and autophagy-associated protein expressions in tumor tissues at the 20th day after intravenous injection of ZrO_2_ NPs and ZrO_2_ NPs + NAC. a: Control; b: ZrO_2_ NPs (50 mg/Kg/d) + NAC (80 mg/Kg/d); c: ZrO_2_ NPs (25 mg/Kg/d); d: ZrO_2_ NPs (50 mg/Kg/d). Data are presented as the mean ± SD (n = 3). Compared to the control group: *p < 0.05, **p < 0.01, ***p < 0.001, and ****p < 0.0001.

The balance between Bcl-2 family members which include anti-apoptotic proteins (such as Bcl-2) and pro-apoptotic proteins (such as Bax) determine the fate of a cell (Adams and Cory, 2007). Bcl-2 expression was down-regulated in the ZrO_2_ NPs groups (25 mg/Kg/d, 50 mg/Kg/d) (Figure 6), suggesting that ZrO_2_ NPs could promote apoptosis in HeLa cells. Compared to the ZrO_2_ NPs (50 mg/Kg/d) group, there was relatively milder up-regulation of Bax and the down-regulation of Bcl-2 in the ZrO_2_ NPs + NAC group.

The family of proteases known as the caspases play a key role in apoptosis (Li and Yuan, 2008). Caspases are central to apoptosis as they include both the initiators which are primarily responsible for the beginning of the apoptotic pathway (such as Caspase-2, -8, -9, and -10) and the executors which are responsible for the definite cleavage of cellular components (such as Caspase-3, -6 and -7) (Thornberry and Lazebnik, 1998; Lakhani et al., 2006). ZrO_2_ NPs could down-regulate the expressions of Pro-caspase-9 and Pro-caspase-3. However, the expressions of Caspase-9 and Caspase-3 were up-regulated by ZrO_2_ NPs, suggesting that Caspase-9 and Caspase-3 are activated. Compared to the ZrO_2_ NPs (50 mg/Kg/d) group, there was a milder up-regulation of Pro-caspase-9 and Pro-caspase-3 and a milder down-regulation of Caspase-9 and Caspase-3 in the ZrO_2_ NPs + NAC group, indicating that ZrO_2_ NPs promote apoptosis and NAC inhibits the apoptosis induced by ZrO_2_ NPs.

Translocation of Bax into mitochondria, which initiates the mitochondrial apoptosis pathway and causes the release of Cyt C from the mitochondria (Guo, et al., 2012) prompts the binding of Apaf-1 to Caspase-9, thus activating Caspase-9 (Brentnall et al., 2013) and inducing subsequent cell death (Jiang and Wang, 2004). Compared to the control group, cytoplasmic Cyt C expression was significantly increased by ZrO_2_ NPs, while the up-regulation of Cyt C in the ZrO_2_ NPs + NAC group was relatively weak. Taken together, these results suggest that NAC inhibits the mitochondria mediated apoptosis induced by ZrO_2_ NPs.

ROS are involved in the regulation of a variety of biological processes (Galadari et al., 2017). Increased ROS in cancer cells may eliminate cancer cells via activating various ROS-induced cell death pathways including autophagy (Chen et al., 2017). In addition to apoptosis, autophagy is also a mode of programmed cell death (Liu and Levine, 2015). LC3-II transforming from LC3-I, a hallmark of autophagy (Rabinowitz and White, 2010), participates in autolysosome formation (Boya et al., 2013). As shown in Figure 6, ZrO_2_ NPs decreased LC3-I expression in HeLa cells but increased LC3-II expression, indicating that LC3-I was transformed into LC3-II after ZrO_2_ NPs treatment. In addition, NAC reversed the changes observed with ZrO_2_ NPs treatment by restoring the expression levels of LC3-I and LC3-II to close to those in the control group.

Recent studies suggest that autophagy plays a dual role in determining cell fate. It either functions as a survival mechanism or induce programmed cell death under different cellular stresses (Zhang et al., 2015b). *ATG 5* is a major autophagy gene required for autophagosome synthesis (Mizushima, 2007). Beclin-1 governs autophagosome formation and recruits other autophagy proteins to the pre-autophagosomal membrane (Kihara, et al., 2001). During the expansion of autophagosome membranes, Atg7 activates Atg12 which is transferred to Atg10 and covalently linked to Atg5 (Kihara, et al., 2001). ZrO_2_ NPs could up-regulate autophagy-related proteins, including Atg5, Atg12, and Beclin-1, while NAC inhibited the up-regulation of their expressions in the ZrO_2_ NPs + NAC group. These results suggest that ZrO_2_ NPs promote autophagy in HeLa cells by activating ATGs and upregulating the expression of autophagy related proteins, while NAC inhibits the effect induced by ZrO_2_ NPs.

### Complete Blood Count, Serum Enzyme Levels and Tissue Images

**FIGURE 7:**
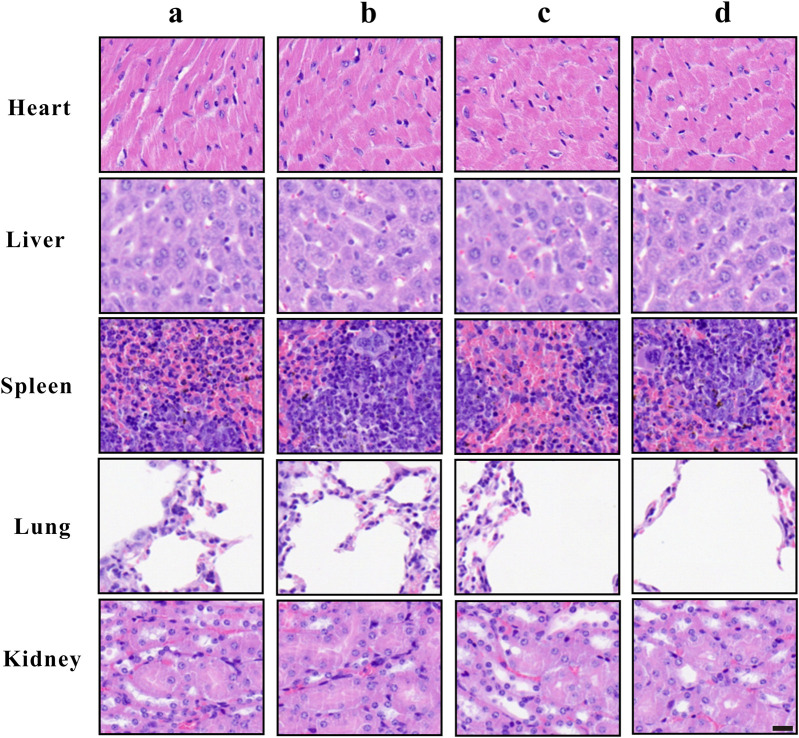
Tissue morphology of heart, liver, spleen, lung, and kidney after ZrO_2_ NPs or ZrO_2_ NPs + NAC treatment, and HE staining. a: Control; b: ZrO_2_ NPs (50 mg/Kg/d) + NAC (80 mg/Kg/d); c: ZrO_2_ NPs (25 mg/Kg/d); d: ZrO_2_ NPs (50 mg/Kg/d). Scale bar: 20 μm.

To assess the hematologic toxicity of ZrO_2_ NPs, CBC analysis was conducted. In mice treated with ZrO_2_ NPs, CBC analysis showed that the counts of white blood cell (WBC), red blood cell (RBC) and platelets (PLT) were all in the normal range. To assess the effect of ZrO_2_ NPs on visceral organ function, serum enzyme assays and histological assays were performed. As shown in Table 1, liver function indicators (alanine transaminase (ALT), aspartate amino-transferase (AST)) did not increase. Renal function indicators (blood urea nitrogen (BUN) and creatinine (Cr)) were not elevated either. Further, ZrO_2_ NPs did not alter cardiac toxicity indexes (lactate dehydrogenase (LDH), hypersensitive troponin T (TNT - HS), creatine kinase (CK), creatine kinase-MB (CK - MB) and myoglobin (Myo)) levels (Table 1). On day 20 after treatment, nude mice vital tissue and organ specimens were sectioned and stained with HE. No significant abnormalities were found in the heart, liver, spleen, lung, and kidney (Figure 7). These results suggest that ZrO_2_ NPs do not cause significant myelosuppression and toxicity to the heart, liver, spleen, lungs, and kidneys. In summary, ZrO_2_ NPs have no apparent systemic toxic effects.

**TABLE 1:** The blood cell counts, the enzyme level and myocardial enzyme spectrum analysis of tumor bearing mice after treated with ZrO_2_ NPs, or ZrO_2_ NPs + NAC.

	Control	ZrO_2_ NPs (50 mg/kg/d) + NAC (80 mg/Kg/d)	ZrO_2_ NPs (25 mg/kg/d)	ZrO_2_ NPs (50 mg/kg/d)
Blood cell count				
WBC(10^9^/L)	7.5 ± 0.4	7.3 ± 0.7	7.5 ± 0.4	7.6 ± 0.4
RBC(10^12^/L)	11.2 ± 1.3	11.3 ± 1.1	11.2 ± 0.7	11.3 ± 1.0
HGB(g/dl)	14.6 ± 0.4	14.7 ± 0.3	14.5 ± 0.5	14.4 ± 0.6
HCT(%)	49.0 ± 1.4	49.2 ± 0.3	48.8 ± 1.1	48.7 ± 0.3
PLT(10^11^/L)	8.6 ± 0.3	8.4 ± 0.5	8.4 ± 0.6	8.3 ± 0.7
Serum enzyme level				
ALT(U/L)	9.6 ± 0.3	9.6 ± 0.4	9.7 ± 0.5	9.8 ± 0.6
AST(U/L)	47.6 ± 2.3	47.7 ± 2.1	48.5 ± 2.5	48.8 ± 2.6
BUN(mmol/L)	2.0 ± 0.2	2.0 ± 0.3	2.2 ± 0.2	2.3 ± 0.3
Cr(μmol/L)	6.6 ± 0.3	6.6 ± 0.4	6.7 ± 0.3	6.8 ± 0.3
Myocardial enzyme spectrum				
TNT-HS(pg/ml)	47.5 ± 3.4	47.4 ± 3.8	47.9 ± 3.1	48.2 ± 3.2
CK(U/L)	3742.0 ± 120.2	3745.3 ± 122.8	3775.4 ± 131.9	3790.3 ± 147.2
LDH-L(U/L)	7695.2 ± 120.2	7699.5 ± 122.3	7695.4 ± 111.8	7709.3 ± 120.4
CK-MB(U/L)	3780.5 ± 76.1	3782.5 ± 101.2	3792.3 ± 104.0	3809.9 ± 112.5
Myo(ng/ml)	73.4 ± 2.8	73.5 ± 3.2	73.7 ± 3.4	73.9 ± 3.5

Data are mean ± SD (*n* = 3).

## Conclusion

**SCHEME 1:**
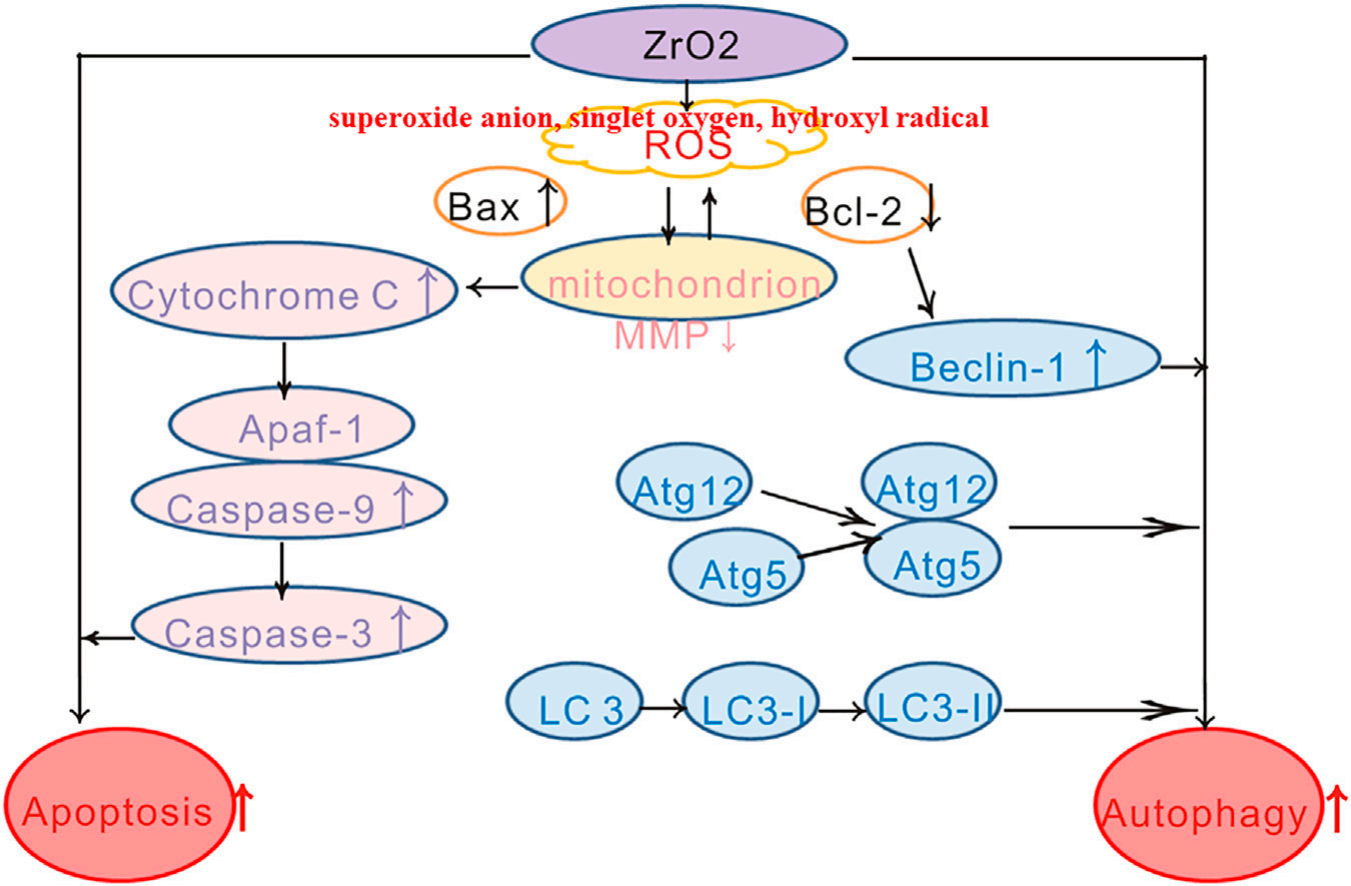
Schematic diagram of HeLa cell death induced by ZrO_2_ NPs through mitochondrial apoptosis and autophagy pathway mediated by ROS.

ZrO_2_ nanoparticles treatment results in swollen mitochondria, increased apoptosis rate, decreased MMP, reduced Ki-67 labeling and increased TUNEL-positive cells, increased expression of mitochondrial apoptotic proteins (Bax, Caspase-3, Caspase-9, and Cyt C). Besides, autophagic vacuoles, increased expression of autophagy-related proteins (Atg5, Atg12, Beclin-1, and LC3-II) can also be detected. To conclude, ZrO_2_ nanoparticles induce HeLa cell death through apoptosis and autophagy pathways. NAC, a ROS-reducing agent, significantly reduced the rate of apoptosis, MMP, and *in vivo* anti-tumor activity. Based on these observations, we conclude that ZrO_2_ NPs could induce tumor cells death via apoptosis and autophagy, which is mediated by ROS (Scheme 1).

## Data Availability

The raw data supporting the conclusions of this article will be made available by the authors, without undue reservation, to any qualified researcher.
