# Supplementary material for: Zirconia Nanoparticles Induce HeLa Cell Death Through Mitochondrial Apoptosis and Autophagy Pathways Mediated by ROS
Source: Front Chem. 2021 Mar 16;9:522708. doi: 10.3389/fchem.2021.522708 (PMC8007972; doi:10.3389/fchem.2021.522708)
Supplement: Supplementary file 1 [file Presentation1.pdf]

Supplementary material

Zirconia nanoparticles induce Hela cell death through mitochondrial apoptosis and autophagy pathways mediated by ROS

Yinghui Shang [a], *Qinghai Wang* [b], *JianLi* [a], *Haiting Liu* [a], *Qiangqiang Zhao* [a], *Xueyuan Huang* [a], *Hang Dong* [a], *Wansong Chen* [c], *Rong Gui* [a*] *and Xinmin Nie* [d*]

[a] Department of Blood Transfusion, the Third Xiangya Hospital, Central South University, Changsha 410013, P. R. China

[b] Department of Cardiology, the Second Hospital of Shandong University, Jinan 250000, P. R. China

[c] College of Chemistry and Chemical Engineering, Central South University, Changsha 410083, P. R. China

[d] Clinical Laboratory of the Third Xiangya Hospital, Central South University, Changsha 410013, P. R. China

[*]Corresponding author.

Phone/Fax: +86-731-8861 8513. E-mail address: aguirong@163.com (R. Gui)

Phone/Fax: +86-731-8861 8577. E-mail address: niexinmin7440@sina.com (X. Nie)

Table of Contents

1. Experimental procedures

1.1 Zeta potentials of ZrO_2 NPs over 180 days

1.2 X-Ray Diffraction (XRD) pattern and crystallinity calculation

1.3 Superoxide anion in HeLa cells detected by Superoxide anion detection kit after treated with ZrO_2 NPs

1.4 Singlet oxygen in HeLa cells detected by singlet oxygen detection kit after treated with ZrO_2 NPs

1.5 Hydroxyl radicals in HeLa cells were detected by hydroxyl radical in situ fluorescence staining kit after treated with ZrO_2 NPs

2. Supplementary figures

1. Experimental procedures

1.1 Zeta potentials of ZrO_2 NPs in PBS (pH 7.4) over 180 days

The ZrO_2 NPs was stored in PBS, and Zeta potentials were measured on day 30, 60, 90, and 180.

1.2 XRD pattern and crystallinity calculation

The ZrO_2 NPs powder was detected by Ultima IV (Rigaku, Japan), with reflection mode. The test range is 5-90 degrees, the test rate is 2 degrees per minute, and the step length is 0.02 degree. The relative crystallinity is calculated by the following formula:

$\varepsilon = Ic/(Ic+Ia)\times 100\%$. Ic is the integral strength of the crystal peak, Ia is the integral strength of the amorphous peak. During the calculation, the diffraction peaks with full width at half maximum (FWHM) greater than 3° were identified as amorphous peaks. The fitting software was JADE. The data were imported into JADE to perform full spectrum fitting of the diffraction peaks within the test range, and the diffraction peaks were described by Pseudo-Voigt function. For the phase with PDF, PDF is directly used for fitting; If not, the straight line is used to find the peak and fit it. Finally, the integral area values of crystallization peak and amorphous peak are obtained, and the crystallinity is calculated.

1.3 Superoxide anion in HeLa cells detected by Superoxide anion detection kit after treated with ZrO_2 NPs

The Bestbio ® BBoxiProbe® Superoxide Anion (O2•-) Detection Kit is a superoxide anion detection kit that utilizes the BBoxiProbe® O88 superoxide anion specific fluorescent probe. BBoxiProbe ® O88 can enter the cell freely through the living cell membrane and is oxidized by the intracellular superoxide anion (O2•-) to produce red fluorescence products. According to the production of red fluorescence in living cells, the amount and change of cell O2•- content can be determined.

The 100 fold diluted probe was added to serum-free cell culture medium, and the cells were incubated at 37℃ for 2 h in dark, washed twice with PBS, observed and photographed under fluorescence microscope (excitation wavelength 518nm, emission wavelength 606nm).

1.4 Singlet oxygen in HeLa cells detected by singlet oxygen detection kit after treated with ZrO_2 NPs

Singlet oxygen specific fluorescent probe R in Singlet oxygen detection kit was used to detect singlet oxygen. Singlet oxygen probe R is a synthetic phenylanthracene fluorescent probe, which

can freely enter cells, react with singlet oxygen in cells, and be oxidized to produce green fluorescent substances. The intensity of green fluorescence is proportional to the level of singlet oxygen in cells, and the changes of singlet oxygen in cells can be known by detecting green fluorescence.

The 100 fold diluted singlet oxygen R probe was added to serum-free cell culture medium, and the cells were incubated at 37℃ for 2 h in dark, washed twice with PBS, observed and photographed under fluorescence microscope (excitation wavelength 488nm, emission wavelength 526nm).

1.5 Hydroxyl radicals in HeLa cells were detected by hydroxyl radical in situ fluorescence staining kit after treated with ZrO2 NPs

Hydroxyphenyl fluorescein (HPF) is a kind of dye that passes through cell membrane freely. Once it reacts with hydroxyl radicals, it generates o-dearylation and fluorescein. These results proved the existence of hydroxyl radical reactive oxygen group in cells. Enhanced green fluorescence indicates high hydroxyl radical content.

Discard cell medium and add 500 mL Reagent A. Dispose of cleaning agent Reagent A, add 500 μl of Reagent B and Reagent C staining solution, incubate with cells in 37℃ cell incubator for 30 min, then discard the staining agent, add 500 μl of preheated Reagent D at 37℃, observe with inverted fluorescence microscope (excitation wavelength 499nm, emission wavelength 515nm) and take pictures.

2. Supplementary figures

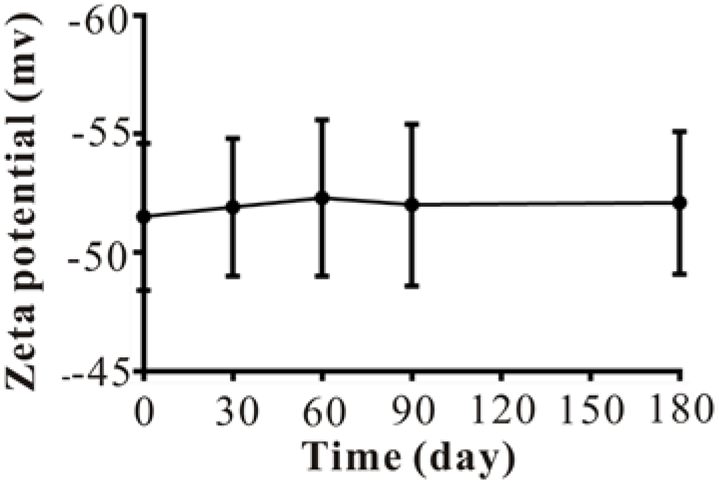

Figure S1. Zeta potentials of ZrO_2 NPs over 180 days. Data are mean ± SD (n = 3).

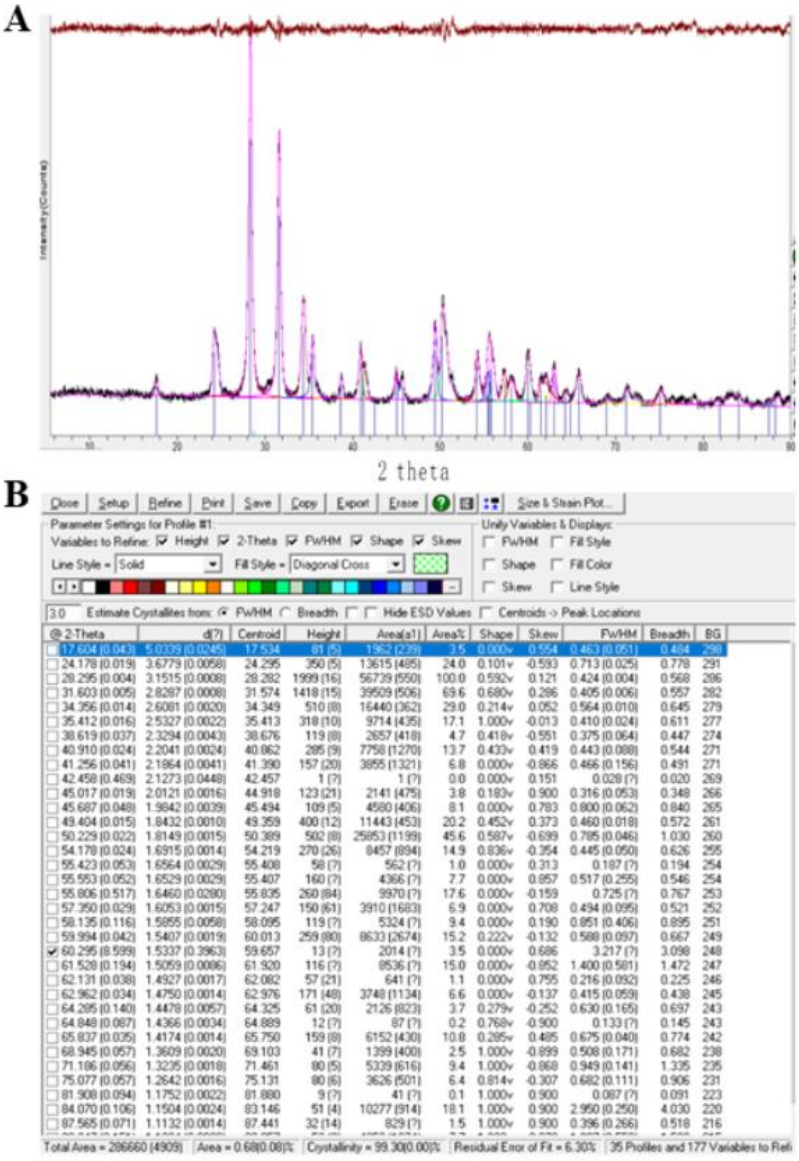

@ 2-Theta	d(?)	Centroid	Height	Area(a1)	Area%	Shape	Skew	FWHM	Breadth	BG
17.604 (0.043)	5.0339 (0.0245)	17.534	81 (5)	1962 (239)	3.5	0.000v	0.554	0.463 (0.051)	0.484	298
24.178 (0.019)	3.6779 (0.0058)	24.295	350 (5)	13615 (485)	24.0	0.101v	-0.593	0.713 (0.025)	0.778	291
28.295 (0.004)	3.1515 (0.0008)	28.282	1999 (16)	56739 (550)	100.0	0.592v	0.121	0.424 (0.004)	0.568	286
31.603 (0.005)	2.8287 (0.0008)	31.574	1418 (15)	39509 (506)	69.6	0.680v	0.286	0.405 (0.006)	0.557	282
34.356 (0.014)	2.6081 (0.0020)	34.349	510 (8)	16440 (362)	29.0	0.214v	0.052	0.564 (0.010)	0.645	279
35.412 (0.016)	2.5327 (0.0022)	35.413	318 (10)	9714 (435)	17.1	1.000v	-0.013	0.410 (0.024)	0.611	277
38.619 (0.037)	2.3294 (0.0043)	38.676	119 (8)	2657 (418)	4.7	0.418v	-0.551	0.375 (0.064)	0.447	274
40.910 (0.024)	2.2041 (0.0024)	40.862	285 (9)	7758 (1270)	13.7	0.433v	0.419	0.443 (0.088)	0.544	271
41.256 (0.041)	2.1864 (0.0041)	41.390	157 (20)	3855 (1321)	6.8	0.000v	-0.866	0.466 (0.156)	0.491	271
42.458 (0.469)	2.1273 (0.0448)	42.457	1 (?)	1 (?)	0.0	0.000v	0.151	0.028 (?)	0.020	269
45.017 (0.019)	2.0121 (0.0016)	44.918	123 (21)	2141 (475)	3.8	0.183v	0.900	0.316 (0.053)	0.348	266
45.687 (0.048)	1.9842 (0.0039)	45.494	109 (5)	4580 (406)	8.1	0.000v	0.783	0.800 (0.062)	0.840	265
49.404 (0.015)	1.8432 (0.0010)	49.359	400 (12)	11443 (453)	20.2	0.452v	0.373	0.460 (0.018)	0.572	261
50.229 (0.022)	1.8149 (0.0015)	50.389	502 (8)	25853 (1199)	45.6	0.587v	-0.699	0.785 (0.046)	1.030	260
54.178 (0.024)	1.6915 (0.0014)	54.219	270 (26)	8457 (894)	14.9	0.836v	-0.354	0.445 (0.050)	0.626	255
55.423 (0.053)	1.6564 (0.0029)	55.408	58 (?)	562 (?)	1.0	0.000v	0.313	0.187 (?)	0.194	254
55.553 (0.052)	1.6529 (0.0029)	55.407	160 (?)	4366 (?)	7.7	0.000v	0.857	0.517 (0.255)	0.546	254
55.806 (0.517)	1.6460 (0.0280)	55.835	260 (84)	9970 (?)	17.6	0.000v	-0.159	0.725 (?)	0.767	253
57.350 (0.029)	1.6053 (0.0015)	57.247	150 (61)	3910 (1683)	6.9	0.000v	0.708	0.494 (0.095)	0.521	252
58.135 (0.116)	1.5855 (0.0058)	58.095	119 (?)	5324 (?)	9.4	0.000v	0.190	0.851 (0.406)	0.895	251
59.994 (0.042)	1.5407 (0.0019)	60.013	259 (80)	8633 (2674)	15.2	0.222v	-0.132	0.588 (0.097)	0.667	249
60.295 (8.599)	1.5337 (0.3963)	59.657	13 (?)	2014 (?)	3.5	0.000v	0.686	3.217 (?)	3.098	248
61.528 (0.194)	1.5059 (0.0086)	61.920	116 (?)	8536 (?)	15.0	0.000v	-0.852	1.400 (0.581)	1.472	247
62.131 (0.038)	1.4927 (0.0017)	62.082	57 (21)	641 (?)	1.1	0.000v	0.755	0.216 (0.092)	0.225	246
62.962 (0.034)	1.4750 (0.0014)	62.976	171 (48)	3748 (1134)	6.6	0.000v	-0.137	0.415 (0.059)	0.438	245
64.285 (0.140)	1.4478 (0.0057)	64.325	61 (20)	2126 (823)	3.7	0.279v	-0.252	0.630 (0.165)	0.697	243
64.848 (0.087)	1.4366 (0.0034)	64.889	12 (?)	87 (?)	0.2	0.768v	-0.900	0.133 (?)	0.145	243
65.837 (0.035)	1.4174 (0.0014)	65.750	159 (8)	6152 (430)	10.8	0.285v	0.485	0.675 (0.040)	0.774	242
68.945 (0.057)	1.3609 (0.0020)	69.103	41 (7)	1399 (400)	2.5	1.000v	-0.899	0.508 (0.171)	0.682	238
71.186 (0.056)	1.3235 (0.0018)	71.461	80 (5)	5339 (616)	9.4	1.000v	-0.868	0.949 (0.141)	1.335	235
75.077 (0.057)	1.2642 (0.0016)	75.131	80 (6)	3626 (501)	6.4	0.814v	-0.307	0.682 (0.111)	0.906	231
81.908 (0.094)	1.1752 (0.0022)	81.880	9 (?)	41 (?)	0.1	1.000v	0.900	0.087 (?)	0.091	223
84.070 (0.106)	1.1504 (0.0024)	83.146	51 (4)	10277 (914)	18.1	1.000v	0.900	2.950 (0.250)	4.030	220
87.565 (0.071)	1.1132 (0.0014)	87.441	32 (14)	829 (?)	1.5	1.000v	0.900	0.396 (0.266)	0.518	216

Total Area = 286660 (4909) | Area = 0.68(0.08)% | Crystallinity = 99.30(0.00)% | Residual Error of Fit = 6.30% | 35 Profiles and 177 Variables to Refi

Figure S2. (A) The XRD pattern and (B) crystallinity of powder ZrO_2 NPs.

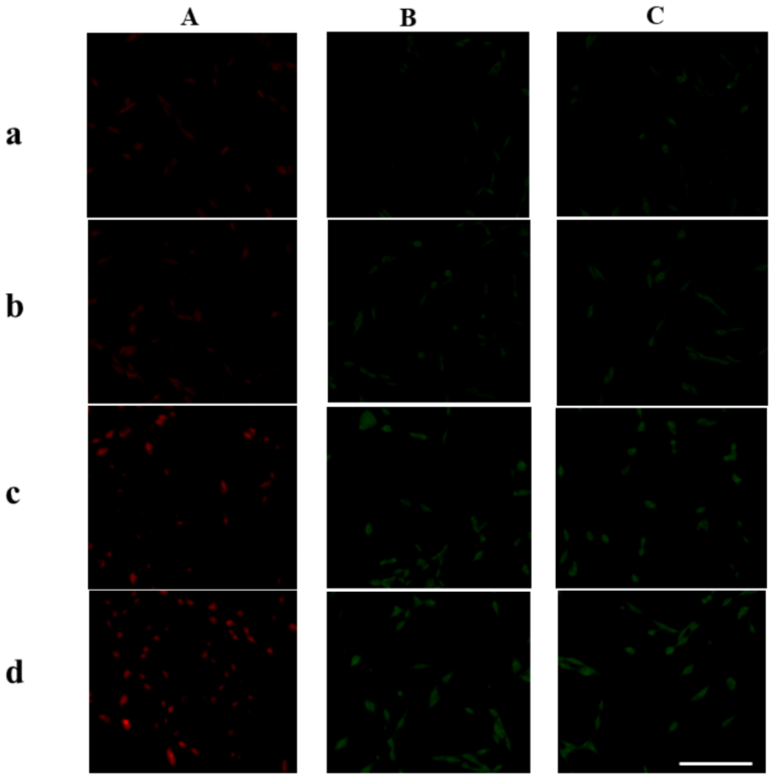

Figure S3. (A) Superoxide anion in HeLa cells detected by Superoxide anion detection kit after treated with ZrO_2 NPs. (B) Singlet oxygen in HeLa cells detected by singlet oxygen detection kit after treated with ZrO_2 NPs. (C) Hydroxyl radicals in HeLa cells were detected by hydroxyl radical in situ fluorescence staining kit after treated with ZrO_2 NPs. a: Control; b: ZrO_2 NPs (100 μg/mL) + NAC (160 μg/mL); c: ZrO_2 NPs (50 μg/mL); d: ZrO_2 NPs (100 μg/mL). Scale bar: 100 μm.
