# Supplementary material for: Zirconia Nanoparticles Induce HeLa Cell Death Through Mitochondrial Apoptosis and Autophagy Pathways Mediated by ROS
Source: Front Chem. 2021 Mar 16;9:522708. doi: 10.3389/fchem.2021.522708 (PMC8007972; doi:10.3389/fchem.2021.522708)
Supplement: Supplementary file 2 [file Table1.DOCX]

Table 1 The blood cell counts, the enzyme level and myocardial enzyme spectrum analysis of tumor bearing mice after treated with ZrO2 NPs, or ZrO2 NPs + NAC. Data are mean ± SD (n=3).

|  | Control | ZrO2 NPs  (50 mg/Kg/d)  + NAC  (80 mg/Kg/d) | | ZrO2 NPs  (25 mg/Kg/d) | | | ZrO2 NPs  (50 mg/Kg/d) |
| --- | --- | --- | --- | --- | --- | --- | --- |
| Blood cell count | | | | | | | |
| \| WBC(10^9^/L) \| \| --- \| | 7.5±0.4 | | 7.3±0.7 | | 7.5±0.4 | 7.6±0.4 | |
| RBC(10^12^/L) | 11.2±1.3 | | 11.3±1.1 | | 11.2±0.7 | 11.3±1.0 | |
| HGB(g/dL) | 14.6±0.4 | | 14.7±0.3 | | 14.5±0.5 | 14.4±0.6 | |
| HCT(%) | 49.0±1.4 | | 49.2±0.3 | | 48.8±1.1 | 48.7±0.3 | |
| PLT(10^11^/L) | 8.6±0.3 | | 8.4±0.5 | | 8.4±0.6 | 8.3±0.7 | |
| Serum enzyme level | | | | | | | |
| ALT(U/L) | 9.6±0.3 | | 9.6±0.4 | | 9.7±0.5 | 9.8±0.6 | |
| AST(U/L) | 47.6±2.3 | | 47.7±2.1 | | 48.5±2.5 | 48.8±2.6 | |
| \| Urea(mmol/L) \| \| --- \| | 2.0±0.2 | | 2.0±0.3 | | 2.2±0.2 | 2.3±0.3 | |
| \| CRE(μmol/L) \| \| --- \| | 6.6±0.3 | | 6.6±0.4 | | 6.7±0.3 | 6.8±0.3 | |
| Myocardial enzyme spectrum | | | | | | | |
| TNT-HS(pg/ml) | 47.5±3.4 | | 47.4±3.8 | | 47.9±3.1 | 48.2±3.2 | |
| CK(U/L) | 3742.0±120.2 | | 3745.3±122.8 | | 3775.4±131.9 | 3790.3±147.2 | |
| LDH-L(U/L) | 7695.2±120.2 | | 7699.5±122.3 | | 7695.4±111.8 | 7709.3±120.4 | |
| CK-MB(U/L) | 3780.5±76.1 | | 3782.5±101.2 | | 3792.3±104.0 | 3809.9±112.5 | |
| Myo(ng/mL) | 73.4±2.8 | | 73.5±3.2 | | 73.7±3.4 | 73.9±3.5 | |

|  |  |  |  |  |
| --- | --- | --- | --- | --- |
